# Epidemiological and clinical aspects of ear nose and throat sensorineural emergencies in the Yaoundé reference hospital

**DOI:** 10.11604/pamj.2016.24.251.7573

**Published:** 2016-07-18

**Authors:** François Djomou, Yves Christian Andjock Nkouo, Eko David Mindja, Choffor Nchinda, Luc Meka, Emilia Mbamyah-Lyonga, Alexis Ndjolo

**Affiliations:** 1 ENT Unit, Yaoundé Teaching Hospital, Yaoundé; 2 Faculty of Medicine and Biomedical Sciences, University of Yaoundé 1; 3 ENT Unit, Yaoundé Reference Hospital, Yaoundé; 4 ENT, Yaoundé Central Hospital, Yaoundé

**Keywords:** Sensorineural, emergency, ENT

## Abstract

**Introduction:**

Sensorineural emergencies (SNE) are rare clinical situations. Few patients consult early explaining subsequent difficulty in having accurate data and management. Three clinical conditions are considered SNE in otolaryngology; they include sudden sensorineural hearing loss (SSHL), Bell's palsy and acute vertigo. There is very little data available on sensorineural emergencies in our setting. The aim of this study was to provide preliminary data on the management of Ear Nose and Throat (ENT) sensorineural emergency cases in Yaoundé Reference Hospital.

**Methods:**

A descriptive retrospective study was carried out based on data collected over a period of 5 years, January 2010 to July 2014 at the Yaoundé Reference Hospital. Information was obtained from patients’ files collected from the archives of the institution. Patients presenting with SSHL, Bell's palsy, acute vertigo who consulted during the study period were included in the study.

**Results:**

A total of 22 patients were included in the study out of 6406 patients who consulted at the ENT Unit. The prevalence of SNE in ENT consultations was 0.003, distributed as follows; 13 patients (59.1%) of SNE had Bell's palsy, seven (31.8%) had vestibular neuritis and two (9.1%) had SSHL.

**Conclusion:**

The prevalence of SNE was low with idiopathic Bell's palsy being the most frequent. There was a general delay in arrival of patients hence delay in diagnosis. This delay could equally be a factor for treatment failure and poor prognosis. More effort should be made in terms of population sensitization about the necessity of getting early medical attention.

## Introduction

Sensorineural emergency cases (SNE) are rare clinical situations. Few patients are seen early during the course of these conditions explaining subsequent difficulties in having accurate data and good management. Three clinical entities are considered sensorineural emergencies in otolaryngology; they include sudden sensorineural hearing loss (SSHL), Bell's palsy and acute vertigo. There is very little data available on sensorineural emergencies in our setting. This study was carried out in order to provide preliminary data on these emergencies in the Yaoundé Reference Hospital. Obtaining data could help improve management of patients presenting this condition. The study objectives were to describe the general profile of patients presenting a SNE in Yaoundé Reference Hospital, to define the epidemiological profile of patients presenting SNE, determine the prevalence of SNE in Ear Nose and Throat (ENT) consultations of Yaoundé Reference Hospital and describe the clinical profile of patients presenting the condition.

## Methods

This was a descriptive retrospective study. It consisted of data collected over a period of 4 years; from January 2010 to July 2014. This study was conducted at the Yaoundé Reference Hospital. Information was obtained from patient files from the archives of the institution. Patients presenting SSHL, Bell's palsy, acute vertigo and who consulted during the study period were included. Files with incomplete data were excluded. SSHL was defined as perceptive hearing loss of greater than 30dB over three contiguous pure-tone frequencies occurring within 72 hours. Bell's palsy was defined as sudden peripheral facial nerve paralysis of unknown origin evolving for less than a week. Vertigo was defined as active acute sustained vertigo found to be of peripheral origin due to dysfunction of the peripheral vestibular system.

Vertigo was defined as active acute sustained vertigo found to be of peripheral origin due to dysfunction of the peripheral vestibular system. Unfortunately in our context we do not have the possibility of realising nystagmography so the diagnosis of vestibular neuritis was purely clinical, based on patient history and clinical exam. For SSHL which was defined as perceptive hearing loss of greater than 30dB over three contiguous pure-tone frequencies occurring within 72 hours, a pure tone audiometric exam was used to pose the diagnosis. Concerning treatment, it was difficult to collect precise data given that a non-negligible proportion of patients started on automedication drugs which they could hardly remember with precision. This could have an impact on the outcome and so we could not affirm that the evolution was solely due to the treatment prescribed by the specialist. Different specialists had different protocols for the same condition. A listing was established from the consultation record book of the ENT unit. All the files of patients received for hypoacusis, vertigo and facial nerve palsy were consulted in an initial phase. Files of patients received for SNE, whose clinical description matched our definitions were retained. Socio-demographic and clinical data were obtained from the files of patients found to match the inclusion criteria. A pre-tested data entry form was used to record information from these patient files. Microsoft Excel^®^ 2013 and EPI INFO^®^ version 3.5.1 software were used for data analysis. Information obtained from files was kept confidential, no names were written on data entry forms.

## Results

*General Profile of Study Population:* a total of 22 patients were included in the study. During the study period, 6406 patients consulted at the ENT unit. Ages of patients included ranged from 16 to 66 years. The mean age was 43.2 ± 17 years (Table 1). The sex ratio was 1.69 with male predominance. Interestingly, there was a general delay in time of consultation for these patients presenting SNE with 64% (14) of patients seeking medical care after 72 hours of evolution. Fourteen percent (3) of patients carried automedication before they arrived at the hospital; 5% (1) consulted a nurse and 23% (5) a general practitioner; 59% (13) of patients were seen by an ENT specialist. The prevalence of SNE in ENT consultations during the study period was 0.003 and was distributed as follows; 13 patients (59.1%) had Bell's palsy; seven patients (31.8%) had vestibular neuritis and two patients (9.1%) had SSHL

**Table 1:** Demographic characteristics of patients

Age group	Frequency	Percentage (%)
10 – 19	3	14
20 – 29	2	9
30 – 39	7	32
40 – 49	2	9
50 – 59	3	14
>59	5	22
**Total**	**22**	**100**

*Bell's palsy:* the prevalence of Bell's palsy in our study population was 0.002. The mean age of patients was 37.5 ± 15 years and sex distribution of patients presenting Bell's palsy showed male predominance (53.8%). Out of the 13 patients with SNE, 8 presented right palsy while the rest had left palsy. All patients were treated on out-patient basis with corticoids and physiotherapy. There was a complete recovery rate of 77%. The majority of patients had palsy of moderate severity (Table 2).

**Table 2:** Grading on House-Brackmann scale of patients with Bell’s palsy

Grade	Frequency	Percentage
2	1	8
3	7	54
4	3	23
5	2	15
**Total**	**13**	**100**

*Vestibular neuritis:* vestibular neuritis occurred with a prevalence of 0.001. The mean age of patients was higher than the mean of those who presented Bell's palsy (52.3 ± 14 years). The sex ratio was 1.75 with male predominance. Concerning clinical aspects, 33% of these patients presented nystagmus and 50% deviation on vestibular examination. Five of the seven patients were hospitalised due to severity of symptoms. All these patients recovered.

*Sudden Sensorineural Hearing Loss (SSHL):* two patients were recorded as SSHL. This gave a prevalence of 0.0003. These two cases were males, aged 38 and 59 years each. They presented perceptive hearing loss on physical examination. Pure-tone audiometric examination confirmed the nature of the hearing loss and showed hearing loss of moderate severity in both cases. They were treated on an out-patient basis. Unfortunately these cases showed no amelioration after a year of evolution.

## Discussion

**General Profile of Study Population:** The attitude of patients concerning initial management was inappropriate only 59% of patient consulted general practitioner.

**Bell's palsy:** The prevalence of Bell's palsy in our study population was 0.002. This was comparable to the results obtained by Katusic et al. in the U.S who found an annual incidence of 20 cases per 100.000 [1]. There was a complete recovery rate of 77%. Adour et al. found in a series of 1000 patients a similar complete recovery rate of 71% [2, 3]. This could be explained by the fact that most patients were seen late in the course of the condition. This permitted the disease to evolve before treatment was instituted.

**Vestibular neuritis:** Vestibular neuritis occurred with a prevalence of 0.001. Newman-Toker et al. in 2008 found a prevalence of vestibular neuritis of 0.002 in US emergency department visits [4]. This higher mean age was similar to that found by Beydilli et al. in 2012 which was 51 years [5]. All these patients recovered, probably by compensation in most cases. Mandala et al. in 2008 [6] also found amelioration in most patients after a week of treatment.

**Sudden Sensorineural Hearing Loss (SSHL):** This gave a prevalence of 0.0003. Shaia et al. in the US found an annual incidence of 5-20 cases per 100,000 [7]. The particularly low prevalence in our study could be due to delay of patients in seeking medical care taking into account the fact that patients seen after 72 hours were not included as SSHL in this study. These two cases were males, aged 38 and 59 years each. The result was comparable to the median range in an American study which was 40-54 years [7]. Recovery rates from studies consulted vary from 47% to 63%. No conclusion can however be drawn from our study with respect to evolution considering the small number of cases. It was noted in the course of the study that treatment protocols were not harmonized; each specialist had their protocol for the different clinical entities of SNE. As a result, recovery rates could have been impacted by inadequate or insufficient treatment. There was a general delay in arrival of patients hence diagnosis. This could explain the extremely low prevalence in our study. This delay could equally be a factor for treatment failure and poor prognosis. More effort is still required in terms of population sensitization about the necessity of getting early medical attention. The study showed some limitations. The retrospective nature of the study with the ever-present problem of missing data from files proved to be a setback. There is very little information on this topic in our setting; consequently there were few studies with which meaningful comparisons could be made. Also the small sample size made it difficult to draw strong conclusions.

## Conclusion

The prevalence of SNE was low with idiopathic Bell's palsy being the most frequent. There was a general delay in arrival of patients hence delay in diagnosis. This delay could equally be a factor for treatment failure and poor prognosis. More effort should be made in terms of population sensitization about the necessity of getting early medical attention. We proposed the harmonization of treatment protocols among all practitioners.

### What is known about this topic

- Sensorineural emergencies are rare clinical conditions. Available literature shows annual incidences of 20 cases per 100.000 for Bell's palsy, 5-20 cases per 100.000 for SSHL and a prevalence of 0.002 in US emergency department visits;
- Patients affected are mostly adults in their forties, usually in a context of multiple health issues linked to age. This makes management sometimes tricky;
- Evolution is favorable in most cases; early diagnosis and treatment are vital for proper management, quick, and complete recovery.

### What this study adds

- Information on the prevalence and the different clinical forms of SNE in our context;
- It brings out challenges faced due to late arrival of patients at medical facilities. This could explain difficulties in management and relatively lower recovery rates;
- It underlines the importance of larger scale studies to draw strong conclusions and elaborate recommendations concerning management in a harmonized manner.
